# Recent Trends in Graphene-Based Sorbents for LC Analysis of Food and Environmental Water Samples

**DOI:** 10.3390/molecules28135134

**Published:** 2023-06-30

**Authors:** João V. B. Borsatto, Fernando M. Lanças

**Affiliations:** Laboratory of Chromatography, Institute of Chemistry at Sao Carlos, University of Sao Paulo, P.O. Box 780, São Carlos 13566-590, Brazil

**Keywords:** liquid chromatography, graphene-based sorbents, sample preparation, food analysis, water analysis, graphene, stationary phase

## Abstract

This review provides an overview of recent advancements in applying graphene-based materials as sorbents for liquid chromatography (LC) analysis. Graphene-based materials are promising for analytical chemistry, including applications as sorbents in liquid chromatography. These sorbents can be functionalized to produce unique extraction or stationary phases. Additionally, graphene-based sorbents can be supported in various materials and have consequently been applied to produce various devices for sample preparation. Graphene-based sorbents are employed in diverse applications, including food and environmental LC analysis. This review summarizes the application of graphene-based materials in food and environmental water analysis in the last five years (2019 to 2023). Offline and online sample preparation methods, such as dispersive solid phase microextraction, stir bar sorptive extraction, pipette tip solid phase extraction, in-tube solid-phase microextraction, and others, are reviewed. The review also summarizes the application of the columns produced with graphene-based materials in separating food and water components and contaminants. Graphene-based materials have been reported as stationary phases for LC columns. Graphene-based stationary phases have been reported in packed, monolithic, and open tubular columns and have been used in LC and capillary electrochromatography modes.

## 1. Introduction

**Figure 1:**
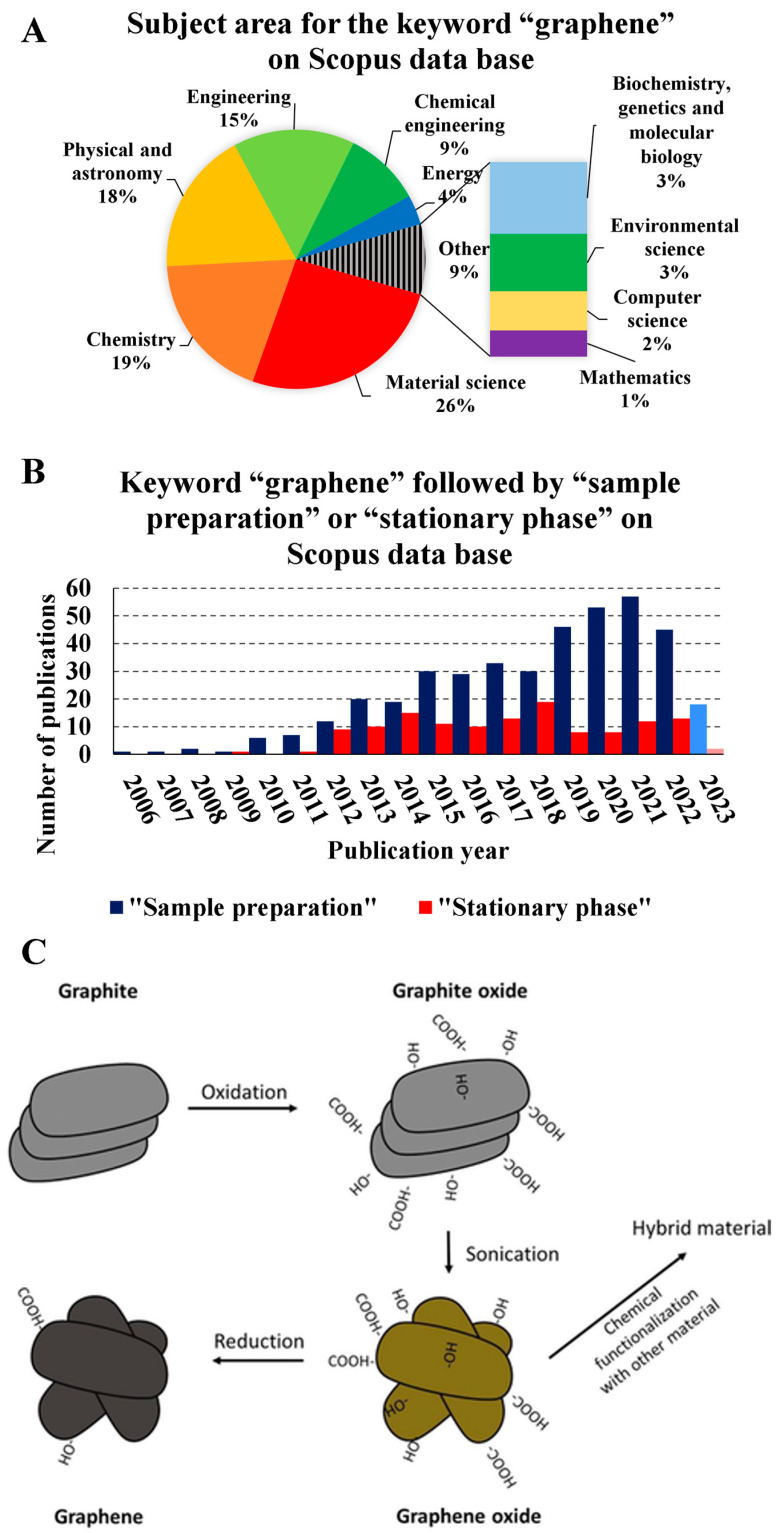
(**A**) Subject area for the keyword “graphene” on the Scopus database. (**B**) The number of publications per year in the database from the keyword search “graphene” followed by “sample preparation” or “stationary phase” on the Scopus database. (**C**) General route to obtain graphene-based materials. Figure 1C is reproduced with permission from J. Sep. Sci., 41, de Toffoli, A.L.; Maciel, E.V.S.; Fumes, B.H.; Lanças, The Role of Graphene-Based Sorbents in Modern Sample Preparation Techniques, Pages 288–302, Copyright (2018), John Wiley and Sons [24].

Graphene and graphene derivates are emerging materials in research and development [1], including in material science, chemistry, physics, and many other fields (Figure 1A). Graphene-based materials mainly comprise SP2 hybridized carbons organized in a hexagonal flat web formed by σ covalent bonds and parallel π bonds, the base graphene structure [2]. Although graphene and its derivatives are relatively new materials, they have a great diversity of applications in analytical chemistry due to their intrinsic characteristics [2]. Among such characteristics, the high electrical conductivity, mechanical resistance, low density, and high light scattering yield can be highlighted, making it an excellent candidate for applications in sensors and detectors [3]. Furthermore, its large surface area also results in a high adsorption capacity, making it a promising material for catalysts and sorbents [4,5]. Due to these characteristics, graphene-based materials have gained prominence in the last decade in sample preparation and chromatography (Figure 1B). The literature presents reviews that cover the physico-chemical characteristics of graphene and graphene-based materials, as well the application of those materials [6,7,8].

Despite the potential for graphene and its derivates as a highly suitable material, the manufacturing obstacle hinders its adoption on an industrial scale. The mechanical exfoliation of graphite through adhesive tape is a practical way to produce graphene-based materials [9,10]. This procedure, also called the Geim–Novoselov scotch method, involves an adhesive tape, which is pressed onto a piece of graphite and lifted off, taking a layer of graphite with it [11]. The process is repeated several times to obtain thinner and thinner layers of graphite. The mechanical force applied by the tape causes the layers to separate, leaving behind clean, smooth surfaces [10]. However, this process is highly dependent on human manipulation, and is laborious. Because of these limitations, efforts have been directed to develop alternative routes to produce large quantities of high-quality graphene-based materials [12]. Among the alternative routes, chemical exfoliation, such as Hummer’s method, provides exciting results, being a practical and relatively less manual way of obtaining this material [13,14,15,16,17]. Also, other methods to produce graphene and graphene-based materials have been described in the literature [18]. Chemical vapor deposition [19,20], electrochemical exfoliation [21,22], and unzipping carbon nanotubes [23] are examples of alternative routes to produce graphene and its derivates. Though different preparation procedures for graphene-based materials are described in the literature, they usually follow a general route for application as sorbents [24]. Firstly, the graphite material is oxidated to form graphite oxide. After the graphite oxide has been obtained, this material is sonicated to separate the layers that compose the graphite oxide, forming graphene oxide (GO) sheets. The GO obtained produces reduced graphene oxide or is functionalized with diverse bindings (Figure 1C).

The diversity of potential functionalizations of graphene-based materials allows for a wide range of applications in separation science. These applications include the use of techniques such as stir bar sorptive extraction (SBSE) [25,26], dispersive solid phase extraction (d-SPME) [27,28], fiber solid phase microextraction (Fiber-SPME) [29,30], in-tube solid-phase microextraction (IT-SPME), and others [2,24] in sample preparation methods. Additionally, graphene-based materials have been explored as packing materials for analytical columns in LC, including using graphene oxide (GO) fixed to silica particles functionalized with C18, graphene (G) fixed to silica particles modified with gold nanoparticles, and reduced graphene oxide (rGO) coated with cellulose fixed to silica particles [31,32,33,34]. The variety of analytes that can be analyzed using these phases is as diverse as applying graphene-based sorbents in analytical techniques. Proteins [35], hormones [30], aromatic contaminants [36], pharmaceutical compounds [37,38], and others [2,39] are examples of analytes separated in columns composed by the graphene-based stationary phase. 

The present review investigates the applications of graphene-based materials in food and water analysis, including their utilization as sorbents for sample preparation and analytical columns for LC, in a time frame from 2019 until 2023. The escalating necessity for enhanced analytical processes in food and water analysis has stimulated interest in using graphene-based materials as efficient and trustworthy solutions. Therefore, this review article aims to illuminate the current state and potential prospects for graphene-based sorbents in food and water analysis.

## 2. Offline Sample Preparation for Liquid Chromatography

Offline sample preparation techniques are the most common strategy in the analytical chemistry field. They consist of preparing the sample for analysis while disconnected from the instrument or application. Some advantages of this strategy are the simple procedure, which is usually not dependent on complex instrumentation set-up, and the possibility of analyzing sample fractions prepared with different analytical techniques. This section summarizes offline extraction techniques in which graphene-based sorbents have been employed recently.

### 2.1. Graphene-Based Materials as Dispersive Sorbents

Dispersive solid phase extraction (d-SPE) and microextraction (d-SPME) are examples of sample preparation techniques that involve graphene-based materials as dispersive sorbents [40]. This sample preparation procedure involves (i) adding a solid phase extraction material, usually a powder, to the matrix, (ii) stirring the suspension, (iii) removing the extraction phase from the matrix, (iv) washing the extraction phase material to remove the residue of the matrix in the solid phase particles surface, and (v) eluting the analytes from the extraction phase with a suitable solvent before analysis.

Graphene-based materials have been employed in d-SPE and d-SPME, and mainly supported in magnetic nanoparticles [41]. Magnetic particles are helpful because they allow a magnet to remove the solid phase material from the matrix without filtration [41]. Furthermore, magnetic graphene-based d-SPME sorbents present an interesting advantage over non-magnetic graphene-based d-SPME sorbents. When inserted into an aqueous sample, the graphene-based materials tend to accumulate due to the hydrophobic characteristics of the graphene sheets, reducing their total surface area and, consequentially, reducing their absorption capacity [41]. The presence of the magnetic particles minimizes the accumulation and benefits the adsorption capacity of the extraction phase [41]. Different methods of preparing graphene-based magnetic sorbents are reported in the literature; they can be divided into three main routes. The first route to be cited is physical adsorption [42]. In this route, the graphene-based material and the magnetic particles are produced separately, then mixed in a solution under stirring or sonication. The graphene sheets are then fixed over the magnetic particles by physical adsorption [42]. This method is simple to perform but usually produces single-use sorbents. Another interesting route to produce magnetic graphene-based sorbents is via the in situ growth of magnetic materials [43]. This method is based on the co-precipitation of magnetic particles and graphene-based material sheets [44]. Also, the solvothermal method is a possibility for this route [45]. Covalent bonding is the third possible route to produce graphene-based magnetic particles [46]. In this method, the graphene is covalently bonded to the magnetic particles by modifying them with linker groupers, such as silane groups, binding them to the graphene-based material [47]. The application of d-SPME and d-SPE graphene-based sorbents is also a trend in food and water analysis. For example, the d-SPE phase composed of magnetic graphene oxide (GO) tert-butylamine (TBA) fixed in the magnetic particles’ (GO/Fe_3_O_4_/TBA) nanocomposite was successfully applied in the determination of herbicides in vegetables and water samples [48]. The GO/Fe_3_O_4_/TBA sorbent was prepared by co-precipitating GO and Fe_3_O_4_ particles. After the obtention of the GO/Fe_3_O_4_ particles, they were functionalized with TBA using ethylene glycol to form covalent bonds. A d-SPME method using the GO/Fe_3_O_4_/TBA to quantify 2,4-dichlorophenoxyacetic acid presented limit of detection (LOD) and limit of quantitation (LOQ) values lower than 0.02 μg·mL^−1^, and the recovery range was between 88.0 and 94.0%. Another graphene-based magnetic phase that has been successfully applied to the analysis of contaminants in vegetables is the GO-Fe_3_O_4_ nanoparticles functionalized with maltodextrin (MD) and β-cyclodextrin (β-CD), which was used for the investigation of triazole and triazine pesticides in corn, tomato, and potato [49]. Co-precipitating the GO with the Fe_3_O_4_ magnetic particles produced this magnetic extraction phase. After the obtention of the GO-Fe_3_O_4_, this material was functionalized with MD and β-CD using epichlorohydrin as a linker. The LODs obtained for determining the pesticides in the vegetables ranged from 0.01 to 0.08 μg·L^−1^ and recovery was between 88.4 and 112.0%. Metal-organic frameworks (MOF) have also been used to functionalize graphene-based sorbents. MOFs are porous crystalline materials formed by assembling metal ions and organic ligands with large surface areas, high porosity, and controllable pore structures. As a recent example, in d-SPME, Fe_3_O_4_ particles functionalized with GO sheets and UiO-66 (a Zr-based MOF) have been used as a sorbent for the determination of food dyes in soft drinks, candies, and pastilles [28]. This interesting phase was produced by co-precipitating GO and Fe_3_O_4_, followed by the addition of ZrOCl_2_·8H_2_O and terephthalic acid into a GO-Fe_3_O_4_ suspension in order to produce the magnetic GO/UIO-66 sorbent. The s-SPME method employing the GO/UIO-66 sorbent presented LODs less than 0.218 ng·mL^−1^, LOQs lower than 9.368 ng·mL^−1^, and recovery ranging from 95.01 to 106.33%. The interaction mechanism between the MOF-based sorbent and the food dye analytes was attributed to multiple mechanisms, including n–π/π–π interactions, Yoshida/dipole–dipole hydrogen bonds, and electrostatic interactions. Though magnetic particles are the primary support for sample preparation in d-SPME and d-SPE in food and water analysis, no magnetic materials have been used. For example, GO-coating polystyrene (PS) microspheres were applied to extract bisphenol endocrine disruptors in environmental water samples [27]. To produce GO-PS sorbents, PS colloid templates were first activated to present a positively charged surface. This material was reacted with a suspension of GO in order to form the desired extraction phase. This GO-PS sorbent was employed in the d-SPME method to determine bisphenol A, bisphenol B, bisphenol AF, and tetrabromobisphenol A, presenting LODs between 0.02 and 0.11 μg·L^−1^ and LOQs of 0.07–0.37 μg·L^−1^ and recovering in a range from 71.1% to 104.8%. The retention mechanism of this GO-PS extraction phase was attributed to the π–π interactions and hydrogen interactions between the sorbent and the analytes. Another interesting variation of the d-SPME using graphene-based sorbents applied in food analysis is the “rotating flat surface solid phase microextraction (RFS-SPME)” method. This strategy consists of suspending and stirring foams of the stationary phase, instead of powder material, in the matrix, and after the end of the stirring the foam can be removed with forceps [50]. The RFS-SPME sample preparation method was employed to analyze sulfonamides in animal-based food using an innovative material, the 3D GO/La fixed in Ni foams [50]. To produce the sorbent, GO and lanthanum nitrate were mixed and sonicated in water, followed by the immersion of nickel foam pieces in order to form the extraction phase. The 3D GO/La Ni foams could be applied in the RFS-SPME sample preparation method, which presented LODs lower than 0.14 µg·L^−1^, LOQs lower than 0.475 µg·L^−1^, and recoveries over 90.0% The evaluated 3D GO/La Ni foams’ extraction phase presented an extraction mechanism sorbent–sorbate combining π–π interaction, hydrogen bonding, and electrostatic interaction.

### 2.2. Graphene-Based Materials as Coating Sorbents

Coating techniques involve sample preparation strategies where the sorbent is coated over, typically, a non-sorptive surface. For example, stir bar sorptive extraction (SBSE) and fiber solid phase microextraction (fiber-SPME) are techniques in which the sorbent is fixed over an inert surface. Usually, the sample preparation process using these techniques involves (i) immersing the device coated with sorbent in a matrix solution, (ii) stirring the solution for a specific time, (iii) removing and washing the device to remove the matrix, and finally (iv) desorbing the analyst in an appropriated solvent before analysis.

SBSE and related techniques are simple and effective ways to extract analytes from the matrix. SBSE devices are usually produced by chemically or physically adhering the sorbent to an inert material [51]. Graphene-based materials have been explored in SBSE using this strategy [26,52]. For example, a Ni bar coated with reduced graphene oxide (rGO) was recently used to evaluate benzotriazole ultraviolet absorbents from environmental water [26]. The SBSE bar was produced through covalent bonding between GO and the Ni foam, forming the rGO-Ni foam sorbent. The formed material was then shaped in a stir bar format. This device could be used as an SBSE sample preparation method to determine the analytes at LODs of 0.33–0.50 μg·L^−1^ and recovery in a range of 83% to 112%. The rGO-Ni SBSE bar presented an attributed extraction mechanism based on π–π interaction and hydrophobic interactions [26]. Moreover, SBSE bars can be produced by covering cheap magnetic materials, such as coating a wire with sorbent. Graphene-based materials have been used to prepare SBSE devices using this strategy. For example, an SBSE bar produced with stainless steel (SS) wire covered with graphene oxide frameworks (GOF) was employed to extract and analyze dyes in water samples [52], presenting LODs of 0.15 to 0.3 ng·mL^−1^, LOQs of 0.5 to 1.0 ng·mL^−1^, and a recovery range of 89.38–108.54%. To produce this device, it was first necessary to activate the SS wire surface to allow the chemical binding of the GO. To do that, the SS wire was submitted to a sequence of reactions until the polydopamine-SS wire was formed. The GO was suspended in a solution and then reacted with the polydopamine-SS wire forming the GOF-SS wire SBSE bar [52]. Another way to produce SBSE bars is by magnetic fixation of the sorbent to the stirring bar [25]. This bar type is used in a sample preparation technique called stir bar sorptive-dispersive microextraction (SBSDμE). As the name suggests, this technique introduces the coated magnetic bar into the sample solution, and under fast stirring the sorbent is dispersed in the solution. After the end of the stirring, the magnetic bar attracts the sorbent forming against the coated bar, which can be removed by plastic forceps [25]. A recent application of this technique using graphene-based materials for water analysis was the use of an SBSDμE device composed of a neodymium stir bar coated by graphene (G) fixed on Fe_3_O_4_ particles’ sorbent (G-Fe_3_O_4_) in the determination of seven pesticides in water [25]. The magnetic G-Fe_3_O_4_ particles were produced by the co-precipitation method, similar to that previously described in the d-SPME section. This SBSDμE method presented a recovery range between 20% and 75%, with a LOQ between 5 ng·mL^−1^ and 9.5 ng·mL^−1^. Another interesting sample preparation technique based on the sorbent phase coating a surface was fiber solid phase microextraction (fiber-SPME). Fiber-SPME consists of the sorbent coating a capillary’s inner or outer surface, forming the extraction fiber. Commonly, fiber-SPME devices are filled with monolithic extraction phases, including the ones modified with graphene-based materials. Graphene-based materials as fiber-SPME sorbents have also been explored for food and water LC analysis in the last five years. For example, a composite prepared with GO, MOF zeolitic imidazolate framework 8 (ZIF-8), and molecularly imprinted polymers (MIP) deposited in fiber was used to evaluate sterol and steroid hormones in white meat, egg yolks, and vegetables [30]. The fiber-SPME device was prepared by first producing the GO-MOF sorbent via the solvothermal method. The GO-MOF material was then reacted with a functional monomer (methacrylic acid), a cross-linking agent (ethylene glycol dimethacrylate), and an initiator of the imprinting polymerization (azo (bis)-isobutyronitrile) inside a capillary in order to form the MIP-GO-MO fiber. Applying this fiber-SPME device in the sample preparation method resulted in LODs ranging from 3 to 5 ng·L^−1^ and recovery ranging from 95.0% to 101.0%. This sorbent presented a mixed interaction mechanism, primarily attributed to the MIP efficient imprinting effect and the differences in size, functional group type, and position of the analytes and polar interactions, hydrogen bonds, and electrostatic interactions [30]. Another interesting application of graphene-based sorbents in food analysis is the combination of multiple fibers for the sample preparation procedure [29]. Using multiple SPME fibers increases the surface area of the extraction device, and consequently enhances the extraction. In food and water analysis, a multifiber-SPME device consisting of G embedded in a poly 4-vinylpyridine-co-ethylene glycol dimethacrylate (VP-co-EGDMA) monolith was used to extract and determine phenoxy acetic acid herbicides in water and rice samples [29]. LODs lower than 0.66 μg·L^−1^ and LOQs lower than 2.27 μg·L^−1^ were obtained, and a recovery of range between 70.0 and 117% was reported using this sample preparation method.

Hollow fiber solid phase microextraction (HF-SPME) is a variation of the fiber-SPME in which the sorbent coats the inner wall of a capillary, forming an open channel in the device’s core [53]. Unfortunately, graphene-based sorbents applied on HF-SPME has been underexplored in food and water LC analysis in the last five years. Though underexplored, a recent example can be found in the literature, comprising an LC analysis of food and water samples. An HF-SPME device was prepared by using a MIL-101(Cr) MOF linked to GO and fixed in a polypropylene (PP) fiber to investigate organophosphorus compounds in tomato, cucumber, and agricultural water samples [54]. The hydrothermal method produced MIL-101(Cr) MOF linked to GO. Lately, the material formed has been immobilized on the wall of the PP fiber. Applying the HF-SPME method in determining organophosphorus compounds in vegetables presented LODs lower than 0.27 μg·L^−1^ and LOQs lower than 0.91 μg·L^−1^. This extraction phase presented an interesting proposed interaction mechanism in which hydrogen bonding participates. However, the adsorption process also influences the interaction of π-electrons of the analyte with the Cr and back donation from the sorbent material into the molecular anti-bonding π∗ orbital [54].

### 2.3. Graphene-Based Materials as Packed Sorbent

Packed sorbent techniques are sample preparation techniques that use a solid adsorbent material packed in a cartridge, a column, or a pipette. The sample preparation procedure of these techniques comprises the following steps: (i) equilibrate the sorbent by flushing a suitable solvent through the cartridge, (ii) load the sorbent with the sample by flushing it through the cartridge (for SPE), (iii) wash the sorbent to remove the matrix, and (iv) elute and collect the analytes using a proper solvent.

Sorbents packed on cartridges are a usual approach for producing SPE devices. Silica particles are frequently used as a support for graphene-based sorbents due to their capacity to provide a stable structure and prevent blockages in cartridges [54]. Additionally, suppose that unbonded graphene, GO, or reduced (rGO) sheets are packed. In that case, they can accumulate and block the SPE device. When graphene-based sheets are bonded to Si particles, the covalent bond prevents the accumulation of free graphene-based sheets and avoids blocking. For example, GO sheets covalently bonded to Si particles (SiGO) and modified with β-CD were packed in a cartridge and used for the extraction and analysis of polycyclic aromatic hydrocarbons (PAHs) from fried food [55]. This strategy resulted in an LOD range of 0.1–0.3 μg·L^−1^ and a recovery range from about 55 to 90%. This stationary phase presents a complex interaction mechanism between the sorbent and the analytes due to the participation of π–π stacking, hydrophobic interaction, and size complementarity interactions. Another interesting graphene-based material packed in an SPE sample preparation cartridge is a zwitterionic sorbent modified with graphene (G) and fixed over Si particles [56]. This sorbent is prepared based on three steps. A sol solution is prepared to form the substrate in the first step. In the second step, the G is added to the previously formed material, forming a sol-gel G composite monolith. In the third step, this material is submitted to a synthesis route to produce the zwitterionic sorbent modified with (G). This combination of materials allowed intermolecular and interionic interactions such as dipole–dipole, ion exchange, and π–π interactions. The zwitterionic sorbent modified with the graphene (G) SPE cartridge was used to determine benzothiazoles, benzotriazoles, and benzenesulfonamides contaminants in environmental water [56], presenting LODs and LOQs lower than 20 ng·L^−1^ and a recoveries range from 48 to 85%. Though graphene-based materials are usually fixed over support, SPE cartridges can be packed with graphene sheets that are not fixed in support. As mentioned above, this kind of packing presents limitations, such as blocking the frits caused by the accumulation of the graphene sheets, but it is still possible. For example, it was reported that rGO sheets packed on SPE cartridges were used to extract aflatoxins from food samples [57]. It was possible to obtain LODs lower than 0.83 ng·g^−1^, LOQs lower than 2.83 ng·g^−1^, and relative recoveries ranging from 70 to 113% using this cartridge. Another interesting example of an SPE sorbent not supported in particles is 3D reduced graphene oxide (3D-rGO), a self-supported material [58]. In this configuration, the rGO material formed a 3D framework with a high content of carbon at the surface and some residual oxygen-containing groups. This sorbent was formed via hydrothermal reaction, using GO as a starting material. The 3D-rGO SPE devices were employed to determine diclofenac in water and effluent samples, presenting a recovery of about 80% [58]. A sorbent–sorbate interaction based mainly on π–π interactions and possibly on hydrogen bonding interactions was attributed to the 3D-rGO sorbent.

Pipet tip SPME (PT-SPME) can be considered a variation of the SPE because the sorbent packed on a pipet tip forms a device similar to a cartridge. Si particles have also been used as a sorbent support for PT-SPME sample preparation. As a recent example, GO sheets fixed on Si particles have reportedly been packed in PT-SPME devices and applied to determine herbicides in sugarcane-derived foods [59]. The SiGO particles were prepared via covalent bonding between the GO and the Si particle, as is usual for this phase. This method showed acceptable LODs of 1.0–5.0 ng·mL^−1^ for juice and 5.0–25.0 ng·g^−1^ for candy and syrup. Additionally, recoveries ranging from 48 to 69%, 34 to 89%, and 28 to 76% were obtained for juice, candy, and syrup, respectively. MOF materials modified with graphene-base materials have also been reported as sorbents for PT-SPME. Si microspheres covalently bonded to fluorinated graphene (FG) and functionalized with ZIF-8 nanocrystals were packed in a PT-SPME device and applied to determine chlorophenols in tap water, honey, and black tea [60]. About 83.7% to 97.7% were for the chlorophenol compounds, with LOQs of 4.76 µg·kg^−1^ and LOQs of 15.9 µg·kg^−1^. This extraction phase presented multi-interaction mechanisms, including π-stacking and hydrophobic and hydrogen-bonding interactions between the adsorbent and the compounds. Though Si particles are standard, other types of particles can be used as supports for graphene-based materials in PT-SPME and have been applied in LC analyses of foods. For example, the nanocomposite GO-starch-polyacrylamide was packed in PT-SPME devices and used to determine antibiotic residues in cow’s milk [14]. The sorbent was produced by copolymerizing GO and polyacrylamide using CCO_3_ to form the porous material. The PT-SPME method presented LODs of 2.7–5.0 μg·kg^−1^ and a recovery range of 88–102%. This extraction phase presented an adsorption mechanism mainly based on hydrogen bond interactions between the analytes and the sorbent [14].

### 2.4. Trends in Graphene-Based Offline Sample Preparation

In the last five years, graphene-based sorbents have successfully been employed in diverse sample preparation techniques for food and water analysis. Water, milk, vegetables, cereals, and meat are examples of the diversity of samples that graphene-based sorbents could prepare recently (Table 1). The diversity of the variety of the matrix represents the diversity of analytes that graphene-based sorbents can analyze. Pesticides, dyes, toxins, drugs, and hormones can be extracted using graphene-based sorbents. Dispersive solid phase extraction and packed sorbent devices, such as SPE cartridges and PT-SPE, are the most common applications of graphene-based sorbents. It has also been observed that GO and rGO sheets are the most usual type of graphene-based material used for the sorbents, and Hummer’s method is the most usual way to obtain graphene-based material. Usually, graphene-based sorbents for sample preparation are prepared with no functionalization, or functionalization with MOF. The supporting material of the graphene-based sorbents is also important because it directly affects the application of those materials. In dispersive solid phase extraction, magnetic supports are receiving the most attention; for coated techniques (SBSE and fiber-SPME), a variety of supports has been presented; and for packed columns, Si particles have been the most-used support in recent years.

## 3. Online Sample Preparation for Liquid Chromatography

**Figure 2:**
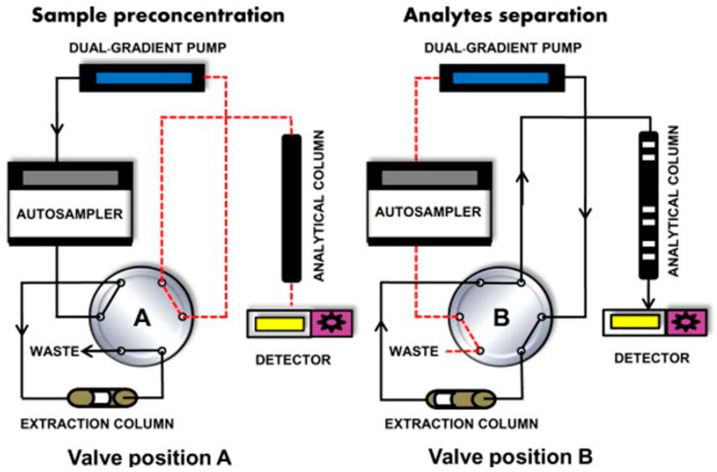
General scheme of a column switching (online IT-SPME) liquid chromatography approach. Valve position A represents the extraction procedure, and valve position B represents the elution and separation procedure. Reproduced with permission from TrAC Trends Anal. Chem., 62, Fernández-Ramos, C.; Šatínský, D.; Šmídová, B.; Solich, P., Analysis of Trace Organic Compounds in Environmental, Food and Biological Matrices Using Large-Volume Sample Injection in Column-Switching Liquid Chromatography, Pages 69–85, Copyright (2014), Elsevier [63].

The online sample preparation strategy presents a significant advantage over offline techniques; the automation process minimizes the chance of human error, one of the most common error sources in analytical methods [61,62]. Though it has advantages over offline techniques, the need for more expensive and specialized equipment limits its wide adoption [62,63]. In-tube SPME (IT-SPME), also referred to as a synonym of column-switching, is the most-used strategy for online sample preparation in LC. This technique consists of (i) loading the extraction column with the samples with the help of a weak mobile phase (usually pure water), while a valve directs the flow after the extraction column to waste, (ii) stopping the load and switching the valve to connect the extraction column to the analytical column (second column), and (iii) starting the elution and LC separation. Figure 2 shows a regular online IT-SPME analysis.

Graphene-based materials fixed over silica particles are an interesting sorbent for this application. For example, GO supported into the Si particles’ (SiGO) sorbent was packed inside a fused silica capillary to produce a miniaturized extraction column employed in the extraction of β-lactam antibiotics from environmental water samples [13]. This method presented LODs lower than 0.3 μg·L^−1^ and recovery between 70.4 and 91.6%. In-tube SPME also utilized SiGO particles’ functionalization with other bindings. For example, SiGO particles functionalized with C18 and end-capped were used for the online sample preparation of coffee samples, targeting the determination of xanthines [64]. LOQs of about 0.3 to 1.0 µg·L^−1^ and recoveries between 73 and 109% were obtained. Both works above employed a similar route to prepare the SiGO particle or its functionalized derivate with C18 and end-capping. Briefly, GO sheets, produced by Hummer’s method, were covalently bonded to the Si particle’s surface. For the functionalization with C18 and end-capping, the SiGO particles formed were reacted with chlorodimethyl-n-octadecylsilane and trimethylchlorosilane in separate steps [64]. Monoliths can also be a support for graphene-based materials for IT-SPME. A simple way to produce graphene-based monoliths is to suspend graphene-based materials in a polymerization mixture and insert it into the extraction column hardware [65]. After polymerization, the graphene is fixed together in a monolithic structure. An example of graphene-based monoliths’ applications in online IT-SPME analysis in foods is the use of GO incorporated in an ethylene glycol dimethacrylate (EDMA) monolithic column to extract and analyze 16 sulfonamides in chicken muscle and milk samples [65]. The in situ polymerization of Go and EDMA inside a 10 mm × 2.1 mm stainless steel capillary produced the monolithic column. LODs of 0.3 μg·kg^−1^ for milk and 0.6 μg·kg^−1^ in chicken muscle and recoveries of about 70.3 to 98.5% and 79.0 to 108.0% for each matrix, respectively, were obtained [65]. Coated bars inserted inside LC tubing are also an alternative for preparing IT-SPME devices [66]. An interesting recent example of this strategy is to apply a device composed of stainless steel (SS) wire coated with SiGO mesoporous structure and place it inside a PEEK tube to analyze PAH in honey samples [66]. This IT-SPME device was produced by coating a wire with epoxy resin and attaching the sorbent powder material. The SiGO mesopore material was prepared with a hydrothermal reaction [66] This IT-SPME approach could detect PAH from honey, which presented a LOD of 0.25 ng·g^−1^.

Headspace in-tube solid phase microextraction (HS-SPME) using a graphene-based sorbents is another sample preparation strategy that has been applied recently, hyphenated to LC. This strategy consists of introducing the sample in a vail and inserting the HS-SPME device into the space above the liquid surface; after the extraction procedure, the HS-SPME system is connected online to LC equipment. A recently reported application of graphene-based sorbents in the HS-SPME technique for food analysis was performed, utilizing a stainless steel tubing coated with a composite formed by GO and ionic liquid (IL) for the analyses of the headspace of naphthalene, a volatile PAH, in honey samples [67]. The GO-IL sorbent was prepared by reacting GO and 1-methyl imidazole. The prepared material was then electrochemically deposited on an SS tube, forming the HS-SPME device [67]. The sample preparation method presented a LOD of 0.1 ng·mL^−1^, a LOQ of 0.3 ng·mL^−1^, and a recovery range between 90.0 and 106.5%.

In short, a trend exists to employ GO as the graphene-based sorbent in online sample preparation (Table 2). The fixation of the GO sheets in Si does not allow the movement of the sheets, and the sequential blocking of the column is caused by the accumulation of the GO sheets in the column frit. These characteristics make the packing of extraction columns with graphene-based phases viable. As mentioned in the previous section, wide possibilities for functionalizing GO materials are available, and some of them are interesting for online approaches. There is space for advancements using specialized materials in IT-SPME and HS-SPME, and their exploration might result in significant improvements in the detection of contaminants in food and water samples. Though IT-SPME is the most popular approach for online sample preparation in liquid chromatography, graphene-based materials are also possible in other techniques, such as the needle-sleeve-based online hyphenation of solid-phase microextraction and liquid chromatography [68]. However, to our knowledge, these approaches have not been applied to food and environmental water analysis.

## 4. Stationary Phase for Liquid Chromatography

**Figure 3:**
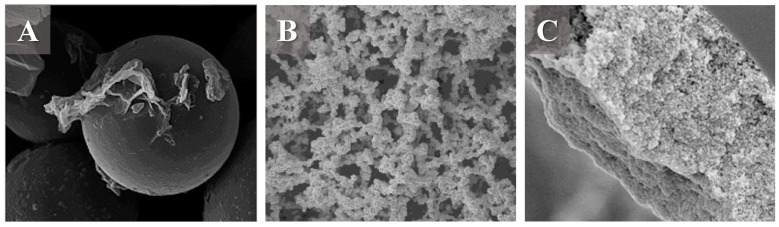
(**A**) Example of the SiGO-C18ec sorbent used as a sorbent for a packed column. Reproduced with permission from J. Chromatogr. A, 1685, Borsatto, J.V.B.; Maciel, E.V.S.; Lanças, F.M., Investigation of the Applicability of Silica-Graphene Hybrid Materials as Stationary Phases for Capillary Liquid Chromatography., Starting page 463618, Copyright (2022), Elsevier [71]. (**B**). Example of the poly (GO-co-TAIC-co-MMA) sorbent used as a sorbent for a monolithic column. Reproduced with permission from J. Chromatogr. B, Anal. Technol. Biomed. Life Sci. 1203, Guo, Y.; Si, H.; Li, H.; Zhao, X.; Zhao, Y.; Li, S.; Wang, Q.; Zhu, B., Graphene Oxide-Based a Network Porous Poly (Trially Isocyanurate-Co-Methacrylate) Monolithic Column for HPLC Separation of Aromatic Molecular and Lipopeptide Antibiotics., Starting page 123310, Copyright (2022), Elsevier [38]. (**C**). Example of the poly (MGONPs) sorbent used as a sorbent for an open tubular column. Reproduced with permission from J. Chromatogr. A, 1667, Şeker, S.; Alharthi, S.; Aydoğan, C., Open Tubular Nano-Liquid Chromatography with a New Polylysine Grafted on Graphene Oxide Stationary Phase for the Separation and Determination of Casein Protein Variants in Milk., Starting page 462885, Copyright (2022), Elsevier [35].

Graphene-based materials have also been used as stationary phases for liquid chromatography columns. These sorbents have been employed in packed, monolithic, and open tubular (OT) columns, the three most common types of LC columns (Figure 3). These sorbents have also been used in capillary electrochromatography (CEC), a hybrid between LC and capillary electrophoresis [69,70]. This section discusses the application of graphene-based sorbents as a stationary phase for separating usual food and environmental water contaminants in packed, monolithic, and OT columns in LC and CEC.

### 4.1. Packed Columns

Packed columns are the standard types of columns in LC. They comprise particles of the stationary phase packed inside a tubing (or capillary tubing). This type of column is easy to produce given its production procedures demand only a pump that allows the packing of the stationary phase in the column hardware [72]. The simplicity of the production makes packed columns a straightforward strategy for evaluating new stationary phases. Graphene-based sorbents are in an early development stage as a stationary phase for LC column, but some examples are present in the literature [34,73,74]. Usually, graphene-based materials, such as GO sheets, are fixed over silica particles once it is easily the packing procedure [34,73,74]. GO is the most present in the LC column among the graphene-based materials [31,32,73,74], but graphene quantum dots (GQD) are also commonly used in graphene-based sorbents [75,76,77,78,79]. Diverse functionalizations of GO sheets are reported in the literature. β-CD-functionalized graphene-based materials are interesting materials being explored when focusing on separating chiral mixtures [78,80]. Other functionalizations with gold [31], IL [81], C18 [71], and cellulose [32] are examples reported in the literature.

Graphene-based packed LC columns are still underexplored for food and water analysis. However, some applications reported in the last five years are described in the literature. In seed samples, a capillary column packed with a SiGO-C18ec stationary phase, produced by the functionalization of SiGO particles with C18 followed by end-caping (as previously mentioned), was employed to separate lecithin, a common phospholipid component in foods. This study evaluated the viability of applying an online liquid extraction (OLE) system coupled with an LC-MS to evaluate the hyphenated extraction and separation of lecithin from the pounder of seeds [82]. The SiGO-C18ec column presented a better separation than a conventional C18 column in reversed-phase (RP) separation mode for this application. Graphene-based packed LC columns have also been used to separate contaminants in food and water. For example, sulfonamides, frequently detected antibiotics in food and water samples [83], have recently been the focus of separation studies utilizing graphene-based stationary phases. LC columns packed with porous graphene (PG), fixed over Si particles, have been employed for the separation of sulfanilamide, sulfamethazine, sulfamerazine, sulfasalazine, sulfadiazine, and sulfamethoxazole in HILIC mode [37]. PG materials are graphene-based materials with random or highly stable carbon atom vacancies in the sheet [37]. To produce the PG-Si sorbent, (3-aminopropyl)triethoxysilane is used to link the PG material to the Si particles. Sulfonamides have also been separated in HILIC mode using GQD fixed on Si particles (Si-GQD) modified with octadecyl amine and serine; this column has also separated nucleosides in HILIC mode [84]. To prepare this material, the GQD, produced by the solvothermal method, was factionalized with octadecylamine and serine to present hydrophilic and hydrophobic groups in its structure. Lately, the functionalized GQD has been bonded to Si particles using (3-isocyanatopropyl)triethoxysilane as a linker [84]. The same column could also be applied for the RP separation mode, which effectively separated alkylbenzenes and PAHs [84]. Another interesting application of the GQDS-derived material is the application of poly(N-isopropyl acrylamide) (PNIPAAm) functionalized Si-GQDs particles as a stationary phase for the multiclass separation of water-soluble compounds, including contaminants, alkylbenzenes, PAHs, biphenyls, nucleosides/nucleobases, phenols, and anilines [76]. The material was produced by functionalizing Si particles with (3-Aminopropyl)triethoxysilane as a liker. Lately, the Si-GQD particles have been functionalized by the covalent bonding of the PNIPAAm to the GQD. The GQDs were prepared by hydrothermal synthesis using GO as starting material. Also, capillary columns packed with the SiGO-C18ec particles prepared similarly to [82] have been reported to separate a multiclass mixture presenting a different selectivity from the conventional C18 column among the separated compounds, including pesticides such as carbofuran, hexazinone, and clomazone [71].

### 4.2. Monolithic Column

Monolithic columns are another popular type of LC column composed of a monolith of the stationary phase, usually inside a capillary tubing. These columns are, conventionally, produced by the in situ polymerization of polymer-based [85] and silica-based [86] monoliths. In addition, monolithic graphene-based columns have been used in LC and capillary electrochromatography (CEC) columns [85,87]. These types of columns have been reported to be adequate for the separation of small molecules, such as alkylbenzenes, polycyclic aromatics, phenols, and anilines [87,88], and long molecules, such as proteins and peptides [88,89].

Graphene-based monolithic columns have been used in LC and CEC modes, but recent food and water analysis applications present few reported examples. In LC, a monolithic column prepared by the polymerization of 3-chloro-2-hydroxypropylmethacrylate (HPMA-Cl) and ethylene dimethacrylate (EDMA), followed by the fixation of GO, was used recently for the determination of chloramphenicol (CAP), a veterinarian antibiotic, and chloramphenicol glucuronide (CAPG), a metabolite from CAP, in honey and milk samples [15]. Another interesting application of graphene-based monolithic columns evaluated five ingredients in *Schisandra*, a purple-red berry typically used in traditional Chinese medicine [38]. For this study, a column produced by the co-polymerization of GO with triallyl isocyanurate (TAIC) and methyl methacrylate (MMA) was employed to separate schizandrol A, schizandrol B, schisandra A, schisandra B, and schisandra C in reversed-phase LC [38]. The CEC mode has also employed graphene-based monolithic sorbents to separate usual water contaminants. In a recent example, a monolithic column was produced by the co-polymerization of GO with polydopamine (PDA) in the enantiomeric separation of ephedrine and pseudoephedrine isomers [90]. These compounds might contaminate environmental waters [91].

### 4.3. Open Tubular Columns

Open tubular columns have gained popularity in liquid chromatography due to their intrinsic advantages over packed columns, such as low pressures, and their theoretically higher performance [92,93,94]. This type of column presents lower solvent consumption, meeting green chemistry requirements [95]. Additionally, the open channel inside OT columns reduces the multi-path diffusion of the analytes [96]. Open tubular columns consist of a small-diameter capillary, usually coated with a stationary phase. Porous layer open tubular columns (PLOT), wall-coated open tubular columns (WCOT), and bare open tubular columns (BOT) are examples of OT column types [97,98,99]. Open tubular columns were reborn after Karger’s work, and since then they have been widely explored for the separation of large molecules [100,101,102,103].

Nevertheless, the separation of small molecules on OT columns has also been described [96,104,105]. The general procedure of the production of OT columns involves modifying the capillary inner surface with a stationary phase using chemical reaction or physical adsorption methods [106]. Graphene-based OT stationary phases have been employed in LC and CEC separation modes for diverse applications such as pharmaceutical, food, and environmental analysis [35,106,107,108,109].

Graphene-based OT-LC columns have not received much attention for food and environmental water analysis. Very few works explore graphene-based OT columns in food and water analysis. An example reported in the last five years is the separation of casein protein variants from milk samples using an OT column produced with Poly-L-Lysine (PLL) grafted on a methacryloyl graphene oxide nanoparticles (MGONPs) stationary phase [35]. In CEC, examples of graphene-based columns in foods and environmental analysis are more common than in LC, but they are still rare. An interesting example is using molybdenum disulfide and GO composite (GO-MoS_2_) as stationary phases for determining sulfonamides in environmental water [109]. The column was produced by inserting a GO-MoS_2_ dispersion into a fused-silica capillary and leaving it overnight for the GO-MoS_2_ to bond in the fused-silica capillary wall [109]. The separation of other pharmaceutical products that might be found in environmental water has also been reported using graphene-based OT columns in CEC mode [110]. For example, a CEC-OT column produced with a nanocomposite of gold nanoparticles and graphene-carbon nitride was used in the enantioseparation of metoprolol, bisoprolol, propranolol chlorpheniramine, and amlodipine [110]. The enantioseparation of pharmaceutical products in CEC mode has also been reported using the CEC-OT column using GO modified with maltodextrin as a chiral selective phase for the analysis of nefopam, amlodipine, citalopram hydrobromide, econazole, ketoconazole, and cetirizine hydrochloride [107].

### 4.4. Trends Observed in Graphene-Based Stationary Phases in LC Columns

Graphene sorbents have been employed in diverse analytical column configurations in the last five years. Packed, monolithic, and open tubular are the three most common column types, and all present examples are of graphene-based materials as stationary phases (Table 3). In food and environmental analysis, graphene-based stationary phases have been employed in the analysis of food components, such as lecithin, caseins, and schizandrols, and environmental water contaminants, such as antibiotics, alkylbenzenes, PAHs, and others. It was observed that GO is the most usual type of graphene base material for the stationary phase, being most frequently employed with no modifications. While in packed columns Si particles are described as the most common support for GO, and in a monolith organic polymers are the most employed support for the fixation of GO, in the OT column, the GO is directly bonded to the capillary wall or fixed with the assistance of an organic polymer. It can be inferred that the use of graphene-based stationary phases in food and water analysis tends to expand, probably with a diversification of the application of packed columns in LC analysis and OT columns in CEC analysis. In LC, graphene-based sorbents present π–π interactions as the primary separation mechanism [34,75]. For some sorbents and analytes, it is also possible for hydrogen bonding to participate in the interaction mechanism. When aliphatic chains are present in the functionalization of the sorbent, hydrophobic interaction might also participate in the retention process. In CEC, it has also been reported that hydrogen bonding, π–π, and hydrophobic interactions participate in the retention process [70]. GO should remain the preferred choice as the primary component in graphene-based LC stationary phases because of its molecular characteristics, facilitating functionalization. Moreover, the functionalization of graphene-based materials with different bindings tends to increase, finding specific functionalization for specific problems.

## 5. Conclusions

Graphene-based sorbents are becoming more popular year after year in diverse applications. In food and environmental water LC analysis, graphene-based materials have been underexplored in the last five years. However, a trend in the diversification of the application of those materials is also being observed. In offline sample preparation, dispersive solid phase extraction receives the most attention for the application of graphene-based sorbents in food and environmental water LC analysis, but other techniques, such as SBSE, Fiber-SPME, SPE, HF-SPME, and others, could employ graphene-based sorbents with success. Hence, graphene-based sorbents are in an early stages of development, and the number and variety of sample preparation techniques using these materials is tending to increase. In online sample preparation methods, IT-SPME is the most common application of graphene sorbents in food and environmental water analysis. This kind of online sample preparation allows an easy connection to LC analysis, which has been a great advantage of these techniques.

Additionally, the extraction column might be packed with a wide variety of graphene-based sorbents and can deal with a high diversity of samples. Graphene-based sorbents have also been used for analytical LC columns. These sorbents have been utilized in packed, monolithic, and open tubular columns. Graphene oxide bonded to Si particles is the most common graphene-based sorbent used as stationary phases. Graphene-based sorbents have been used as stationary phases in the analysis of food components such as caseins, lecithin, and schizandrols. Graphene-based sorbents have also been employed in separating water contaminants such as alkylbenzenes and antibiotics. The future of graphene-based stationary phases in food and water analyses is promising, with packed columns becoming more frequent for LC and OT columns for CEC analysis. GO should remain the most preferred graphene-based material due to its molecular characteristics, allowing it to be functionalized. Functionalizing these materials with different bindings should be encouraged to find solutions to specific problems.

Graphene-based sorbents present advantages and disadvantages in analyzing food and water samples through liquid chromatography. One significant advantage is the versatility of these sorbents in extracting various analytes while being compatible with different sample preparation techniques. Furthermore, functionalizing GO, and other graphene-based materials, offers a range of possibilities for online approaches. In contrast, as its main disadvantage, these phases are relatively new and may present a lower scope of applications than conventional commercially available extraction phases. In other words, graphene-based materials are not multipurpose, which means that for some applications it is crucial to select an adequate material to extract specific analytes. Another advantage of graphene-based materials is that they can be used as stationary phases in packed, monolithic, and open tubular chromatographic columns. Conversely, detachment followed by the accumulation of graphene sheets in the column outlet can again hinder effectiveness and limit the column’s lifetime. Nonetheless, the functionalization of graphene-based materials with different bindings for specific applications presents intriguing opportunities for improving the diversity of applications for food and water analysis.

## Figures and Tables

**Table 1:** Examples of graphene-based materials in offline sample preparation followed by liquid chromatography analysis since 2019.

Graphene-Based Material	Support	Modification	Techniques	Analytes	Matrix	Year	Ref.
GO	Fe_3_O_4_ particles	TBA	d-SPME	2,4-Dichlorophenoxyacetic acid	Environmental water, lettuce, celery, tomato, and cucumber	2020	[48]
GO	Fe_3_O_4_ particle	MD and β-CD	d-SPE	Triazole, and triazine	Corn, tomato, and potato	2021	[49]
GO	Fe_3_O_4_ particle	MOF/UIO-66	d-SPE	Sunset yellow, tartrazine, allura red	Soft drinks, candies, and pastilles	2023	[28]
GO	PS particle	Not modified	d-SPE	Bisphenol A, bisphenol B, bisphenol AF, tetrabromobisphenol A	Drinking water, tap water, and river water	2022	[27]
GO	Ni foam	La nanoparticles	RFS-SPME	Sulfadiazine, sulfamethoxazole, sulfamethazine	Fresh egg, cow meat, chicken meat, and fish	2022	[50]
GO	SS wire	GOF	SBSE	Sudan G, sudan I, sudan II, and sudan III (dyes)	Water and fruit juice	2019	[52]
GO	Ni bar	Not modified	SBSE	Benzotriazole	Environmental water	2021	[26]
G	Nd bar	Fe_3_O_4_-G	SBSDμE	Boscalid, chlorpyrifos, deltamethrin, dimethenamid-P, dimoxystrobin, metazachlor and tebuconazol	Water	2019	[25]
GO	MIP monolith	MOF/ZIF-8	Fiber-SPME	Sterols: progesterone, testosterone, β-sitosterol, cholesterol, and campesterol	White meat, egg yolks, and vegetables	2019	[30]
GO	VP-co-EGDMA monolith	Not modified	Fiber-SPME	Phenoxyacetic acid, 4-chloro-2-methylphenoxyacetic acid, 2,4-dichlorophenoxyacetic acid, 2-nitrophenoxyacetic acid, and 4-chlorophenoxyacetic acid	Water and rice	2019	[29]
GO	PP monolith	MOF/MIL-101	HF-SPME	Diazinon and chlorpyrifos	Tomato, cucumber, and agricultural water	2020	[54]
GO	Si particles	β-CD	SPE	Benzanthracene, benzofluoranthene, benzo(a)pyrene (bap), anthracene	Fried chicken	2021	[55]
G	Si particles	ZIF-8	SPE	Benzothiazoles, benzotriazoles, benzenesulfonamides.	River water, effluent wastewater, and influent wastewater	2023	[56]
rGO	Not supported	Not modified	SPE	Aflatoxin B1, B2, G1, and G2	Rice and wheat	2019	[57]
rGO	Self-supported	rGO 3D structured	SPE	Diclofenac	Environmental waters	2021	[58]
GO	Starch	Starch	PT-SPME	Amoxicillin, ampicillin, cloxacillin	Milk	2019	[14]
GO	Si particles	Not modified	PT-SPME	Simazine, metribuzin, atrazine, ametryn, tebuthiuron, clomazone, hexazinone, acetochlor, Alachlor, metolachlor, oxyfluorfen	Candy, juice, and syrup	2023	[59]
FG	Si particles	MOF/ZIF-8	PT-SPME	2-chlorophenol, 2,3-dichlorophenol, 2,4-dichlorophenol, 2,5-dichlorophenol, 2,6-dichlorophenol, and 2,4,6-trichlorophenol	Tap water, honey, and black tea	2021	[60]

**Table 2:** Examples of graphene-based materials in online sample preparation followed by liquid chromatography analysis since 2019.

Graphene-Based Material	Support	Modification	Techniques	Analytes	Matrix	Year	Ref.
GO	Si particles	Not modified	IT-SPME	Benzylpenicillin, cefalexin, cefoperazone, and ceftiofur	Water	2023	[13]
GO	Si particles	C18 and end-capping	IT-SPME	Xanthines: theophylline, theobromine, and caffeine	Coffee	2020	[64]
GO	EDMA monolith	Not modified	IT-SPME	Sulfamethoxazole, sulfamoxole, sulfadoxine, sulfamethizole, sulfadimidine, sulfameter, sulfamethoxypyridazine, sulfisoxazole, sulfapyridine, sulfabenzamide, sulfamerazine, sulfamonomethoxine, sulfachloropyridazine, sulfaquinoxaline, sulfadimethoxine, and sulfaphenazole	Milk and muscle	2019	[65]
GO	SS wire	Mesopore Si	IT-SPME	Naphthalene, acenaphthylene, acenaphthene, fluorene, phenanthrene, anthracene, fluoranthene, and pyrene	Honey	2021	[66]
GO	SS tubing	IL/1-methyl imidazole	HS-SPME	Naphthalene	Honey	2020	[67]

**Table 3:** Examples of graphene-based material as a stationary phase for liquid chromatography analysis since 2019.

Graphene-Based Material	Support/Column Type	Surface Modification	Techniques/Separation Mode	Analytes	Matrix	Year	Ref.
GO	Si particles/Packed column	C18 and end-capping	OLE-LC/RP	Lecithin	Seeds	2023	[82]
GP	Si particles/Packed column	Not modified	LC/HILIC	Sulfonamides	-	2019	[37]
GQD	Si particle/Packed column	Octadecylamine and serine	LC/HILIC and LC/RP	Sulfonamides and nucleosides and alkylbenzenes and PAHs	-	2022	[84]
GQD	Si particle/Packed column	PNIPAAm	LC/HILIC and LC/RP	Alkylbenzenes, PAHs, biphenyls, nucleosides/nucleobases, phenols, anilines, water-soluble vitamins, and amino acids	-	2021	[76]
GO	Si particles/Packed column	C18 and end-capping	LC/RP	Carbofuran, clomazone, hexazinone, carbamazepine, citalopram, clomipramine, desipramine, and ochratoxin A	-	2022	[71]
GO	HPMA-Cl and EDMA/Monolith	Not modified	LC/RP	CAP and CAPG	Honey and milk	2022	[15]
GO	TAIC and MMA/Monolith	Not modified	LC/RP	Schizandrol A, schizandrol B, schisandra A, schisandra B, and schisandra C	*Schisandra*	2022	[38]
GO	PDA/Monolith	Not modified	CEC	Ephedrine and pseudoephedrine	-	2019	[90]
GO	MGONPs/OT	PLL	LC/RP	Casein	Milk	2022	[35]
GO	MoS_2_/OT	Not modified	CEC	Sulfisomidine, sulfathiazole, sulfamerazine, phthalylsulfathiazole and sulfacetamide, ulfamonomethoxine and sulfachloropyridazine	Environmental water	2020	[109]
G	Gold nanoparticles/OT	C_3_N_4_	CEC	Metoprolol, bisoprolol, propranolol chlorpheniramine, and amlodipine	-	2021	[110]
GO	Directly coated over fused-silica capillary wall/OT	Not modified	CEC	Nefopam, amlodipine, citalopram hydrobromide, econazole, ketoconazole, and cetirizine hydrochloride	-	2020	[107]

## Data Availability

Not applicable.
